# Canadian Resources on Cannabis Use and Fertility, Pregnancy, and Lactation: Scoping Review

**DOI:** 10.2196/37448

**Published:** 2022-10-19

**Authors:** Ayni Sharif, Kira Bombay, Malia S Q Murphy, Rebecca K Murray, Lindsey Sikora, Kelly D Cobey, Daniel J Corsi

**Affiliations:** 1 Clinical Epidemiology Program Ottawa Hospital Research Institute Ottawa, ON Canada; 2 Faculty of Medicine University of Ottawa Ottawa, ON Canada; 3 Health Sciences Library University of Ottawa Ottawa, ON Canada; 4 University of Ottawa Heart Institute Ottawa, ON Canada; 5 Children's Hospital of Eastern Ontario Research Institute Ottawa, ON Canada; 6 Department of Obstetrics and Gynecology Faculty of Medicine University of Ottawa Ottawa, ON Canada

**Keywords:** cannabis, pregnancy, fertility, breastfeeding, patient education, patient resources, internet, eHealth, digital health

## Abstract

**Background:**

Cannabis use among reproductive-aged Canadians is increasing, but our understanding of its impacts on fertility, pregnancy, and breast milk is still evolving. Despite the availability of many web-based resources, informed decision-making and patient counseling are challenging for expectant families and providers alike.

**Objective:**

We aimed to conduct a scoping review of publicly available web-based Canadian resources to provide information on the effects of cannabis on fertility, pregnancy, and breast milk.

**Methods:**

Following PRISMA-ScR (Preferred Reporting Items for Systematic Reviews and Meta-Analyses extension for Scoping Reviews), we systematically searched 8 databases between January 1, 2010, and November 30, 2020, and web pages of 71 Canadian obstetrical, government, and public health organizations. We included English resources discussing the effects of cannabis on fertility, pregnancy, breastfeeding, or the exposed fetus and infant. Epidemiological characteristics, readability, and content information were extracted and summarized.

**Results:**

A total of 183 resources met our inclusion criteria. Resources included content for public audiences (163/183, 89.1%) and health care providers (HCPs; 31/183, 16.9%). The resources were authored by national-level (46/183, 25.1%), provincial or territorial (65/183, 35.5%), and regional (72/183, 39.3%) organizations. All provinces and territories had at least one resource attributed to them. The majority (125/183, 68.3%) were written at a >10 grade reading level, and a few (7/183, 3.8%) were available in languages other than English or French. The breadth of content on fertility (55/183, 30.1%), pregnancy (173/183, 94.5%), and breast milk or breastfeeding (133/183, 72.7%) varied across resources. Common themes included citing a need for more research into the effects of cannabis on reproductive health and recommending that patients avoid or discontinue cannabis use. Although resources for providers were consistent in recommending patient counseling, resources targeting the public were less likely to encourage seeking advice from HCPs (23/163, 14.1%).

**Conclusions:**

Canadian resources consistently identify that there is no known safe amount of cannabis that can be consumed in the context of fertility, pregnancy, and breastfeeding. Areas of improvement include increasing readability and language accessibility and encouraging bidirectional communication between HCPs and patients.

**International Registered Report Identifier (IRRID):**

RR2-10.1136/bmjopen-2020-045006

## Introduction

### Background

The prevalence of cannabis use in North America is increasing across all age groups as more jurisdictions legalize the production, sale, and possession of nonmedical cannabis products [1,2]. Increases in use are most notable among individuals of reproductive age, including pregnant individuals [3-5]. The recreational use of cannabis was nationally legalized in Canada on October 17, 2018 [6]. Before legalization, the prevalence of self-reported cannabis use among pregnant and recently pregnant individuals was increasing at both the national (adjusted odds ratio 1.18, 95% CI 0.98-1.43) [7] and provincial levels (adjusted relative risk 1.61, 95% CI 1.51-1.72) [3]. Although the data after legalization are limited, further increases are expected [1,8].

A growing body of experimental and epidemiological data suggests adverse effects of cannabis use on reproductive and perinatal health, including on fertility, pregnancy, breast milk, and the exposed fetus or infant [9]. However, the availability of scientific data does not necessarily mean that such data are distributed to, consumed by, or accessible to nonacademic audiences. The public increasingly uses internet resources as a primary source for health information and guidance [10]. Perinatal health information accessed via web-based resources may not be evidence based, up to date, or curated by health care professionals. Furthermore, data suggest public dissatisfaction with the quantity and quality of information available on perinatal cannabis use on the web [11]. Despite the availability of clinical guidelines from obstetrical societies [12,13], many health care providers (HCPs) lack the knowledge or confidence in their ability to provide counseling to their patients about cannabis use [14], including topics related to pregnancy [15]. Recent findings from the United States show that many HCPs do not respond to cannabis use disclosures or offer to counsel [16]. When counseling occurs, it frequently does not extend beyond general statements or discussions regarding potential legal or social services implications. A lack of counseling poses a significant challenge. Patients may infer from an absence of discussion that cannabis use is safe, with no impact on fetal development or later child health [11].

### Objective

Many Canadian organizations may seek to guide perinatal cannabis use through web-based resources. However, the scope, consistency, and accessibility of available resources have not been previously evaluated. Therefore, the objective of this scoping review was to identify and characterize all publicly available web-based educational resources and clinical guidelines that provide information to the Canadian public and HCPs on the short-term and long-term effects of cannabis use on fertility, during pregnancy, and while breastfeeding.

## Methods

### Study Design

The protocol for this scoping review was registered a priori in the Open Science Framework [17] and has been published [18]. Protocol deviations are noted in Multimedia Appendix 1. Our methodology followed established frameworks for scoping reviews [19,20] and involved identifying the research question; identifying relevant literature or resources; selecting literature or resource; charting the data; and collating, summarizing, and reporting the results. Findings were reported in keeping with the PRISMA-ScR extension [21].

### Search Strategy

Our search strategy was developed by a health sciences librarian (LS), with iterations completed in consultation with the study team and subsequently peer-reviewed by a second information specialist using the Peer Review of Electronic Search Strategies guideline [22].

To identify resources targeting the Canadian public and HCPs, we searched the websites of 71 Canadian organizations known to provide information on pregnancy and breastfeeding (federal and provincial health or public health agencies and national and regional obstetrical and perinatal societies and networks; Multimedia Appendix 2). These websites were identified in consultation with stakeholders in our professional networks. Websites were manually searched using a predefined keyword search strategy described in the published study protocol [18]. Resources with publication dates before 2010 were excluded. Those without publication dates were retained. Website search was completed manually by 2 independent reviewers (KB and AS) and validated by a third independent reviewer (MSQM).

To supplement our search for resources targeting HCPs, we also searched medical databases for professional care guidelines, position statements, and clinical recommendations. The search strategy was developed in MEDLINE and then translated into the other databases (Multimedia Appendix 3). We systematically searched MEDLINE and MEDLINE in Process via Ovid, Embase Classic + Embase via Ovid, ERIC via Ovid, CINAHL via EBSCOHost, and Education Source via EBSCOHost from January 1, 2010, to November 30, 2020, a 10-year contemporary sample encompassing the date of national legalization of the sale of nonmedical cannabis in Canada.

### Study Selection

Eligible resources were those that (1) were developed by or on behalf of a Canadian organization; (2) were published in English or French between 2010 and 2020; (3) targeted clinicians or lay public; and (4) provided recommendations, guidance, or reports on the safety or impacts of cannabis use on male or female fertility, pregnancy, the developing fetus, or breast milk and breast milk–fed infants.

There were no limitations on resource formats; thus, eligible resources included web pages, infographics, posters-based resources, video resources, and clinical guidelines or position statements.

### Screening

For records identified via database searching, title, abstract, and full-text screening were conducted using DistillerSR [23] by 2 independent reviewers (KB and AS). Discrepancies arising at each step were discussed until a consensus was reached, and a third reviewer (MSQM) consulted when necessary. Records identified via website searching were assessed against predefined screening criteria as detailed in the published study protocol [18], and the URLs of eligible records were documented. The reference lists of all the included resources were reviewed to identify any relevant records that our search strategy may have missed.

### Charting the Data

For resources published in peer-reviewed journals, we extracted the title, journal name, date of publication, name and email of the corresponding author, and the publishing or authoring organization, group, or society that developed the resource. For resources identified through website searches, we extracted the URL, the document title, date of publication (if available), date accessed for extraction, and the organization, group, or society that developed the resource. In addition, the use of visuals, videos, and references has been documented. Additional extracted characteristics included the availability of resources in languages other than English, the perceived target population (HCPs, general public, and both), contributions from patient partners or the general public, contributions from external organizations, cannabis-related terminology, the scope of the information presented on cannabis use, and recommendations made (if any). The accessibility and readability of the web-based resources were also determined. Readability was assessed using the Simple Measure of Gobbledygook [24]. Accessibility was documented as the reviewers’ perception of how easy it was to find the resource from the parent website’s home page. A resource was subjectively classified as “very easy” or “easy” to find through keyword searches on the parent website. A resource was classified as “not easy” to find if the reviewer was only able to find it after exhausting all possible keyword search strategies or if the resource appeared late in the search result pages (eg, appeared on the 20th search page). As they were not found through manual website searches, resources that were identified via the database search were classified as “not applicable.” The extent to which content on fertility, pregnancy, and breastfeeding was mentioned within each resource was subjectively coded as “core to the document,” “significantly represented,” and “mentioned briefly.”

### Collating, Summarizing, and Reporting the Results

Extracted data were analyzed using quantitative (ie, frequencies and percentages) and qualitative (ie, thematic and exemplar quotes or excerpts) methods. Tables were then created to contextualize the level of jurisdiction of the publishing organization (national, provincial, or regional) and key characteristics and concepts of the included resources. Key characteristics were summarized separately for resources targeting the HCPs and the public. A word cloud was used to visualize the number and frequency of terms used to refer to cannabis and cannabis products [25].

## Results

### Overview

**Figure 1:**
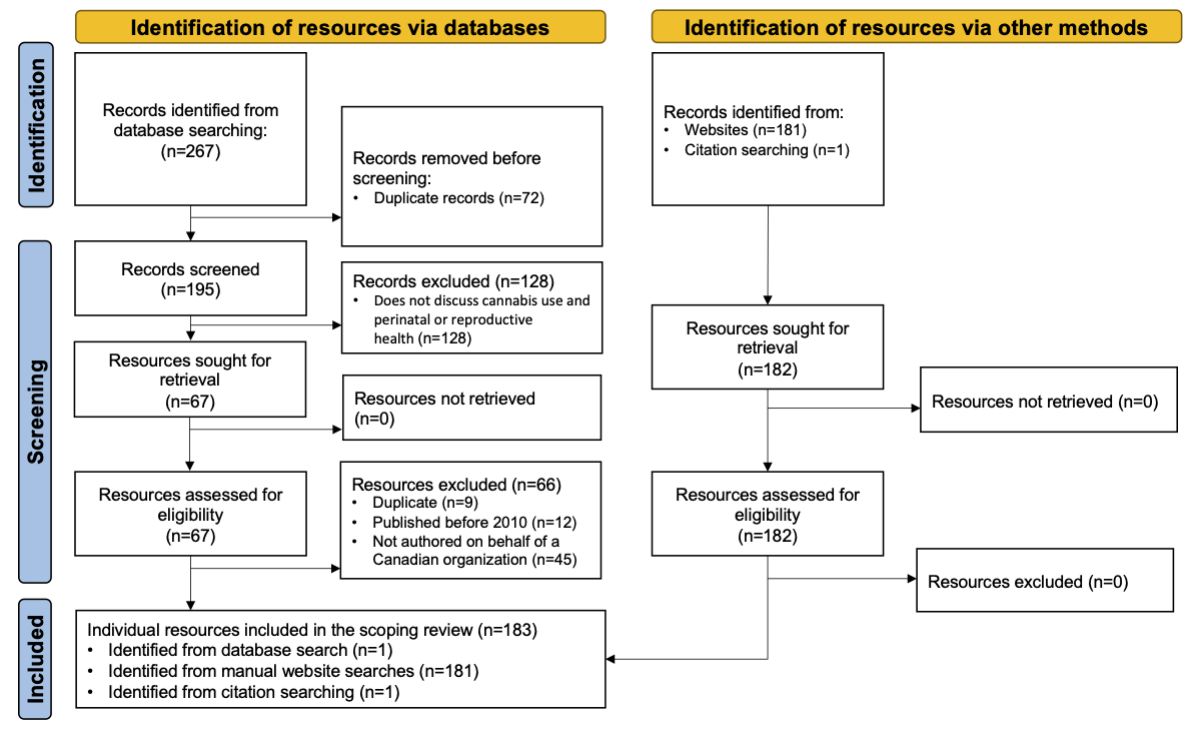
PRISMA-ScR (Preferred Reporting Items for Systematic reviews and Meta-Analyses extension for Scoping Reviews) flow diagram.

Our search strategy yielded a total of 377 articles and resources. A total of 267 records were identified from the database search of which 72 were excluded because they were duplicate records; 28 were excluded through title and abstract screening; and 66 were excluded through full-text screening. In total, 181 resources were identified through manual website searching, and 1 resource was identified through a review of reference lists of the included resources. Thus, 183 resources met the eligibility criteria to be included in the study (Figure 1). The individual characteristics of the included resources are shown in Multimedia Appendix 4.

### Distribution of Resources by Canadian Geography

The included resources came from national-level organizations (46/183, 25.1%), provincial- or territorial-level organizations (65/183, 35.5%), and lower-level regional organizations within provinces and territories (eg, community organizations, regional health authorities, or public health units; 72/183, 39.3%). All 13 Canadian provinces and territories had at least one resource attributed to them. The provinces or territories with the greatest number of published resources (including resources from provincial- or regional-level organizations) were Ontario (72/137, 52.6%), British Columbia (28/137, 20.4%), and Alberta and Quebec (6/137, 4.4% for both; Table 1).

**Table 1:** Summary of educational resources included in this review by the geography of publishing organization.

			Resources, n (%)		Examples of authoring organizations		RefIDs of individual resources^a^
**Authoring organization level of jurisdiction (N=183)**							
	National organization	46 (25.1)		Society of Obstetricians and Gynaecologists of Canada		1, 2, 7-13, 22-24, 26-28, 31, 38-45, 48, 57, 73-76, 84, 137-150, 182	
	Provincial or territorial organization	65 (35.5)		Centre for Addiction and Mental Health		Summarized below by province or territory	
	Regional organization^b^	72 (39.3)		Champlain Maternal Newborn Regional Program		Summarized below by province or territory	
**Authoring organization by home province or territory (n=137)^c^**							
	Alberta	6 (4.4)		Alberta Health Services		3-6, 21, 69	
	British Columbia	28 (20.4)		Perinatal services BC		15-17, 33-35, 50, 54, 61-68, 70-72, 111-118, 127	
	Manitoba	3 (2.2)		Government of Manitoba		77-79	
	New Brunswick	2 (1.5)		Government of New Brunswick		80, 81	
	Newfoundland and Labrador	3 (2.2)		Government of Newfoundland and Labrador		82, 83, 85	
	Northwest Territories	3 (2.2)		Government of Northwest Territories		90-92	
	Nova Scotia	2 (1.5)		Government of Nova Scotia		86, 87	
	Nunavut	2 (1.5)		Government of Nunavut		88, 89	
	Ontario	72 (52.6)		BORN Ontario		14, 18-20, 25, 30, 32, 36, 37, 46, 47, 49, 51-53, 55, 56. 59, 59, 102-110, 119-126, 128-136, 151, 154-156, 159-181, 183	
	Prince Edward Island	4 (2.9)		PEI Chief Public Health Office		29, 93-95	
	Quebec	6 (4.4)		Gouvernement du Québec		58, 60, 152, 153, 157, 158	
	Saskatchewan	5 (3.6)		Government of Saskatchewan		96-100	
	Yukon	1 (0.7)		Government of Yukon		101	

^a^For full citations, see Multimedia Appendix 4.

^b^Includes community organizations, regional health authorities, and public health units.

^c^Excludes resources authored by a national organization.

### Characteristics of Resources on Cannabis Use

#### Overview

Of the 183 resources identified, 15 (8.2%) were published before 2018 (before the national cannabis legalization in Canada), 57 (31.1%) were published in or after 2018, and 111 (60.7%) did not report the year of publication (Table 2). All publication dates were obtained from a manual website search. A total of 16.9% (31/183) of resources included information for HCPs, and 89.1% (163/183) had content specific to the general public. A total of 6% (11/183) of resources included content for both the public and the HCPs. A total of 74.3% (136/183) of resources included references of primary information sources for readers to refer to.

A broad terminology was used to refer to cannabis and its derivative products (n=56). The 4 most-frequently used terms were “cannabis” (n=174 mentions), “THC” (n=107 mentions), “marijuana” (n=63 mentions), and “CBD” (n=62 mentions; Multimedia Appendix 5).

**Table 2:** Characteristics of educational resources included in this scoping review.

Variables			All records (N=183), n (%)		Targeted audience^a^, n (%)		
					Health care providers (n=31)		Public (n=163)
**Year of publication**							
	Before 2018 (year of national legalization)	15 (8.2)		16 (51.6)		10 (6.1)	
	On or after 2018	57 (31.1)		6 (19.4)		47 (28.8)	
	Not reported	111 (60.7)		9 (29)		106 (65)	
Resource is a clinical guideline			3 (1.6)		3 (9.7)		1 (0.6)
Specified contributions from patient partners or members of the public			8 (4.4)		3 (9.7)		6 (3.7)
**Ease of finding the resource^b^**							
	Very easy	107 (58.5)		14 (45.2)		100 (61.3)	
	Easy	49 (26.8)		2 (6.5)		41 (25.2)	
	Not easy	23 (12.6)		5 (16.1)		16 (9.8)	
	Not applicable	2 (1.1)		2 (6.5)		0 (0)	
**Available languages**							
	English	183 (100)		31 (100)		163 (100)	
	French	80 (43.7)		12 (38.7)		75 (46)	
	Another language^c^	7 (3.8)		0 (0)		7 (4.3)	
**Approximate reading grade level^d^**							
	4-6	5 (2.7)		0 (0)		5 (3.1)	
	7-9	40 (21.9)		6 (19.4)		35 (21.5)	
	≥10	125 (68.3)		24 (77.4)		110 (67.5)	
	Not applicable (<100 words)	11 (6)		1 (3.2)		10 (6.1)	
Resource is or includes an infographic			10 (5.5)		0 (0)		10 (6.1)
Resource is or includes a video or videos			29 (15.8)		14 (45.2)		22 (13.5)
Resource includes references			136 (74.3)		14 (45.2)		130 (79.8)

^a^Some records had content targeting both providers and the public and so may be represented in both columns.

^b^Resources were subjectively classified by the reviewer as “very easy,” “easy,” or “not easy” to find through keyword searches of the parent website. A resource was classified as “not easy” to find if the reviewer was only able to find it after exhausting all possible keyword search strategies or if the resource appeared late in the search result pages (eg, appeared on the 20th search page). Resources identified via the database search were classified as “not applicable.”

^c^Other languages included Chinese, Farsi, Korean, Punjabi, Spanish, Vietnamese, Arabic, Farsi, Inuktitut, and Innuinnaqtun.

^d^Measured using the Simple Measure of Gobbledygook [24].

#### Resources for the Public

Of the 163 resources providing information to the public, only 6 (3.7%) specified contributions from patient partners or the public. On the basis of a subjective measure of difficulty to find the resources using keyword searches on the search engines within the parent-organization websites, of the 163 resources, 141 (86.5%) were “easy” or “very easy” to find, and 16 (9.8%) were “not easy” to find. In addition to English, 75 (46%) resources were available in French (an official language of Canada), and 7 (4.3%) were also available in other languages. Over half of the public-facing resources (110/163, 67.5%) were at an approximate 10th grade reading level or higher. A total of 6.1% (10/163) of resources included one or more infographics, and 13.5% (22/163) included one or more videos.

#### Resources for HCPs

Of the 31 resources with content for HCPs, 6 (19%) were published on or after 2018, and 9 (29%) did not report the date of publication. A total of 10% (3/31) of resources were clinical guidelines, and 10% (3/31) specified contributions from patient partners or the public. A total of 52% (16/31) of resources were deemed easy or very easy to find, and 16% (5/31) were not easy to find. A total of 39% (12/31) of resources were available in French, and 45% (14/31) included one or more videos.

### Scope of Content Specific to Cannabis Use and Fertility, Pregnancy, and Breast Milk

#### Overview

The extent to which cannabis use and fertility, pregnancy, and breast milk were discussed varied greatly (Table 3). Of the 183 resources, 57 (31.1%) resources were dedicated specifically to providing information on the impact of cannabis use on fertility, pregnancy, and breast milk, but nearly half (87/183, 47.5%) only briefly mentioned the impact of cannabis use on reproductive health.

**Table 3:** Summary of content covered in the educational resources included in this scoping review.

Content		Included resources, n (%)
**Extent to which content on fertility, pregnancy, and breast milk was discussed in the resource^a^ (N=183)**		
	Core to the document	57 (31.1)
	Significantly represented	39 (21.3)
	Mentioned briefly	87 (47.5)
**Content on fertility**		55 (30.1)
	Female fertility	28 (50.9)
	Male fertility	22 (40)
	Sex-specific effects not specified	24 (43.6)
	Identification of a lack of evidence, data, or information	6 (10.9)
**Content on pregnancy**		173 (94.5)
	Use for nausea in pregnancy	38 (22.0)
	Effect on a woman’s body during pregnancy	35 (20.2)
	Effect on exposure fetus or newborn	117 (67.6)
	Identification of a lack of evidence, data, or information	39 (22.5)
**Content on breast milk or breastfeeding**		133 (72.7)
	Effect on mother’s breast milk	47 (35.3)
	Effect on breastfeeding infant	64 (48.1)
	Identification of a lack of evidence, data, or information	34 (25.6)

^a^Subjectively evaluated based on how much content the resource contained on the topics in question relative to the total amount of information presented in the resource.

#### Content on Fertility

The potential impacts of cannabis use on fertility were identified by 30.1% (55/183) of resources. Of these 55 resources, 28 (51%) and 22 (40%) resources mentioned or discussed the specific impacts on female and male fertility, respectively. The main theme arising from these resources was that cannabis negatively affects the reproductive systems of both males and females. Resources mentioned a correlation between higher cannabis use and decreased testosterone levels and poor sperm quality (including lower sperm count, mobility, and concentration) and warned that cannabis use may be implicated in decreased male fertility and failed pregnancies. Similarly, resources suggested that cannabis use may affect the menstrual cycles of biological females by affecting ovulation, egg quality, and length of the cycle, thereby leading to difficulties in becoming pregnant.

#### Content on Pregnancy and the Developing Fetus

Cannabis exposure during pregnancy was discussed in 94.5% (173/183) of resources. Of these 173 resources, 38 (22%) included information on cannabis use for the treatment of nausea during pregnancy. The potential effects of cannabis exposure on pregnancy and the exposed fetus or newborn were mentioned or described in 20.2% (35/163) and 67.6% (117/163) of resources, respectively. Common messaging includes the fact that tetrahydrocannabinol (THC) can cross the placenta to the growing fetus and accumulate in the fetal fat and brain cells. Resources have cited varying lengths of time that THC could remain in human tissues, ranging from weeks to months. The indicated short-term effects of cannabis use on the body are also wide-ranging. The following exemplar quotes illustrate the information conveyed:

Women who smoke marijuana are at greater risk for a failed pregnancy because the drug can upset the chemical balance necessary for the safe passage of the embryo from the fallopian tube down to the uterus, potentially resulting in an ectopic (tubal) pregnancy or miscarriage.

Licit and Illicit Drug Use during Pregnancy: Maternal, Neonatal and Early Childhood Consequences; Canadian Centre on Substance Use and Addiction

Using cannabis during pregnancy may affect [the mother’s] DNA and genes, which can be passed on to future generations.

Cannabis and Pregnancy Don’t Mix, Poster #2; Society of Obstetricians and Gynecologists of Canada

THC exposure to the fetus was linked to adverse outcomes, including preterm birth, low birth weight, stillbirth, growth restrictions, fetal or neonatal mortality, and congenital malformations, including heart abnormalities. Others mentioned long-term implications such as neurodevelopmental impairments, reduced motor development, and behavioral and learning issues as infants age; for example:

The effects of cannabis exposure during pregnancy may last a lifetime. Childhood: poor memory function, poor problem solving skills, and an inability to pay attention. Adolescence: Increased risk of depression and /or anxiety. Adulthood: Possible substance use.

Cannabis, Pregnancy, and Breastfeeding Infographics; Society of Obstetricians and Gynecologists of Canada

#### Content on Breast Milk and the Breast Milk–Fed Child

Topics related to breast milk and breastfeeding were mentioned or discussed in 72.7% (133/183) of resources. Among these 133 resources, the specific effects of cannabis use on breast milk were mentioned in 47 (35.3%) resources, and the potential effects on breast milk–fed infants were mentioned in 64 (48.1%) resources. General consensus among the resources was that THC could accumulate in the breast milk of lactating individuals using cannabis, and resources suggested that it could be stored in breast milk for up to 2 months. Consequently, resources conveyed that cannabis use during lactation could affect the quality and quantity of breast milk produced; for example:

Marijuana is excreted in your breast milk at levels 8 times higher than your blood marijuana (THC).

Marijuana; The MotHERS Program

Cannabis use may inhibit the production of prolactin and reduce the rate of milk production.

Cannabis use during pregnancy and lactation; perinatal services, BC

Cannabis use can affect the quality and quantity of breast milk you produce. THC is stored in your breast milk for long periods of time.

Cannabis and Your Baby; Chatham-Kent Public Health

The effects of infant exposure to THC through the consumption of breast milk were described to include slower motor development, reduced muscular tone, poor suckling or difficulty latching (harder to feed the infant), and issues with learning or behavior and mental health; for example:

THC (delta-9-tetrahydrocannabinol), the substance in cannabis responsible for the “high”, is found in the breastmilk of women who smoke cannabis. If using cannabis affects your mind and body, it may also affect your child’s mind and body. Like THC, CBD is likely to accumulate in fatty tissues, such as breast tissue.

Is cannabis safe during preconception, pregnancy, and breastfeeding? Government of Canada

#### Identification of a Lack of Evidence, Data, or Information About Cannabis Use and Reproductive Health

Of the 55 resources with content on fertility, only 6 (11%) identified a lack of evidence regarding the effect of cannabis on male or female fertility. Of the 173 resources with content on pregnancy and the developing fetus, 39 (22.5%) identified a lack of information regarding the effect or safety of cannabis on pregnancy or the developing child. Among the 133 resources mentioning breast milk, 34 (25.6%) identified a lack of information regarding the effect of cannabis on breast milk or breastfeeding infants, which is evident from the following example:

Further research is needed to better understand the long‑term health effects of cannabis consumption in any form. Further research is needed to allow people to make better informed decisions.

Cannabis Use During Pregnancy; Canadian Association of Midwives

### Recommendations Made for Cannabis Use and Fertility, Pregnancy, and Breastfeeding

In terms of guidance and recommendations provided by the resources included in this review, the overall theme was that cannabis use should be avoided by individuals who are trying to conceive, those who are pregnant, and those who breastfeed their infants. Therefore, cannabis use for the treatment of nausea and vomiting in pregnancy was not recommended; for example:

Cannabis is not recommended to treat nausea and vomiting during pregnancy. Ask a health care provider about safer options to feel better.

Nausea and Vomiting, KFL&A Public Health

Pregnant and lactating women or individuals were often grouped together as a single population for the delivery of recommendations; for example:

Avoid cannabis completely if you are pregnant or breastfeeding.

Cannabis and Your Health; Government of Canada

Of the 163 public-facing resources, only 23 (14.1%) specifically recommended that patients speak to their HCPs about cannabis use in the context of reproductive and perinatal health. One resource recommended that patients speak to their HCPs if using cannabis and planning a pregnancy; 17 suggested speaking to an HCP for further information on using cannabis during pregnancy and 12 for information on using cannabis during lactation; for example:

Some women are interested in using cannabis during pregnancy to treat nausea or “morning sickness”. There is some research showing that women who use cannabis report relief from these symptoms; however, more research is needed to understand the potential health risks. Talk to your healthcare provider if you have questions about this.

Women and Cannabis; Centre of Excellence for Women’s Health

In contrast, all the content for HCPs advised counseling patients about the risks of cannabis use; for example:

It is prudent to advise pregnant women and women thinking of becoming pregnant of the risks associated with cannabis use during pregnancy. The safest option available to pregnant women is to avoid using cannabis. Experts recommend against using any type of cannabis during pregnancy or breastfeeding.

Clearing the Smoke on Cannabis, Canadian Centre on Substance Use and Addiction

The relationship between prenatal cannabis use and LBW underscores the need for clinical management of cannabis use during pregnancy and lactation. Patients should be asked about cannabis use and advised to discontinue cannabis use during pregnancy and lactation.

Alberta Antenatal Pathway; Maternal Newborn Child & Youth SCN

## Discussion

### Principal Findings

In this scoping review of Canadian resources on cannabis use and reproductive and perinatal health, we found that resources targeting both HCPs and the public consistently recommend avoiding cannabis while individuals are trying to become pregnant and during pregnancy and lactation. Ontario-based organizations authored most of the public-facing resources; most were published in English only and used language above a 10th grade reading level. Few resources cited patient-partner collaborations as part of the development process, and a minority incorporated visual or audio-visual aids. Although HCP resources consistently identified the importance of patient counseling, resources for the public rarely recommended consultation with HCPs.

### Strengths and Limitations

This study provides critical insights into the scope of publicly available information on the effects of cannabis use on fertility, during pregnancy, and while breastfeeding. Our methodology was strengthened by following established frameworks for scoping reviews. In addition, our use of a broad and iterative search strategy developed in collaboration with an information specialist, maternity care experts, and a patient partner further strengthened the yield of possible resources from public health, maternal and child health, and substance use authorities. However, there are relevant limitations that should be acknowledged. First, although our database and gray literature searches were comprehensive, some relevant and contributory resources were missed. For example, although manual searches of target websites were thorough, we may not have identified all eligible resources hosted on a given website. Second, we limited our analysis to Canadian resources; as a result, our observations and recommendations may not be generalizable to resources developed by authorities in other regions. Finally, we were unable to ascertain information on the frequency of use (eg, the number of downloads, web page visits, and sharing on social media) and the date of publication for many web-based resources. Thus, we cannot comment on the extent of resource uptake or how resources were being kept up to date.

### Interpretation

The growing popularity of cannabis among individuals of reproductive age, combined with the recent legalization of nonmedical cannabis products in Canada, has necessitated updating or generating clinical recommendations to support HCPs with patient counseling and public resources to guide informed decision-making. However, the development of such resources has proven challenging. Current data on the potential benefits and harms of cannabis use as well as reproductive and perinatal health are still emerging. The volume of published data on these topics has grown exponentially in the last few years, making it challenging to keep resources up to date with reliable information. Although the uptake of health care resources is difficult to ascertain, their usability is greatly influenced by how and in what format they are disseminated. Easy-to-find health care resources that incorporate interactive content where the audience can tailor the information to their personal health care needs and experiences are more likely to be used [26]. Using audio and visual contents alongside plain text and involving or partnering with patients to codevelop resources are also well-recognized strategies for strengthening content, aligning patient and HCP priorities, and improving eHealth literacy [26-28]. Unfortunately, very few resources that we identified incorporated alternative or complementary modes of information sharing, and most did not cite patient involvement in their development. Finally, web-based health information can act as both an enabler and a barrier to shared decision-making [29]—an essential consideration for the development of health care resources and for HCPs when consulting with their patients [30,31]. Although the HCP resources identified in this review were consistent in their recommendation to provide counseling to patients, few public-facing resources examined in this review explicitly recommended that patients consult with HCPs about cannabis use. Failure to identify HCPs as trusted caregivers in patient-facing resources risks perpetuating common barriers to patient counseling in this area [32-34]. Importantly, although not all individuals who use cannabis in pregnancy can have a substance-misuse issue, pregnancy is an optimal opportunity to provide patient education so that informed decisions can be made. To do so necessitates that HCPs stay well informed on general patient-counseling strategies, including counseling strategies specific to perinatal substance use [35].

The resources included in this scoping review represent critical tools for HCPs and the public regarding counseling and decision-making about cannabis use while planning pregnancy, during pregnancy, and lactation. Although the information presented was thematically consistent, we noted common gaps or oversights in existing resources that could be addressed in the future:

1. The authors of educational resources on this topic should regularly update these resources in line with emerging evidence. In line with this, version dates and references should be included for transparency regarding the presented evidence and its recency.
2. Patient-facing resources should clearly and consistently encourage patients to consult with HCPs if they are considering or continuing cannabis use when planning pregnancy or during pregnancy and lactation.
3. Where resources recommend against cannabis use for the management of specific conditions (eg, nausea, anxiety, and chronic pain), suggestions for alternative options or directions to resources outlining alternative options should be provided.
4. Finally, as web-based resources are widely accessible and are generally the public’s first choice to seek information, efforts should be made to increase resource readability and language accessibility. Overall accessibility could be improved by minimizing the use of technical language and text with high reading grade levels, including videos and infographics, and by translating resources to commonly spoken languages in Canada.

### Conclusions

Canadian resources provide information to the Canadian public and HCPs on the effects of cannabis use on fertility, pregnancy, and breast milk and consistently communicate that there is no known safe amount of cannabis that can be consumed in pregnancy. Therefore, these resources recommend against using cannabis if planning pregnancy, during pregnancy, and while breastfeeding. Despite the availability of these resources, improvements can still be made to enhance their accessibility and encourage uptake. Notably, public-facing resources discussing cannabis use related to reproductive and perinatal health should always encourage consultation with HCPs. They should be updated regularly to ensure that guidance reflects current information.

## Appendix

Multimedia Appendix 1 Deviations from the published protocol.

Multimedia Appendix 2 A list of 71 Canadian organizations whose websites were manually searched to identify eligible resources.

Multimedia Appendix 3 Full database search strategy.

Multimedia Appendix 4 Full citations and individual characteristics of resources included in this scoping review.

Multimedia Appendix 5 Word cloud of terminology used to refer to cannabis. The terminology is displayed with the size of the terms in the diagram corresponding to the frequency of their use. Figure generated using “Word It Out” [24].

## Abbreviations

HCP: health care provider
PRISMA: Preferred Reporting Items for Systematic Reviews and Meta-Analyses
THC: tetrahydrocannabinol
