# Supplementary material for: Canadian Resources on Cannabis Use and Fertility, Pregnancy, and Lactation: Scoping Review
Source: JMIR Pediatr Parent. 2022 Oct 19;5(4):e37448. doi: 10.2196/37448 (PMC9631170; doi:10.2196/37448)
Supplement: Multimedia Appendix 1 [file pediatrics_v5i4e37448_app1.docx]

**Online Resource 1**

*Deviations from the study protocol*

The published protocol indicated that the liberal accelerated approach to screening for titles and abstracts of records retrieved through the database search would be used, whereby all documents causing a conflict would proceed to full-text screening. When implemented, two independent reviewers (KB, AS) screened the title and abstracts independently and if discrepancies in assessment arose, these were discussed until a consensus was reached and a third reviewer (MSQM) consulted when necessary. Second, the published protocol indicated that one reviewer would extract the data, and a second reviewer will validate their work. In practice, two independent reviewers (KB, AS) each extracted half the data and confirmed the others’ findings. Finally, minor changes were made to the data extraction form after pilot testing to increase the usability and flow of the questions.
