# Supplementary material for: Canadian Resources on Cannabis Use and Fertility, Pregnancy, and Lactation: Scoping Review
Source: JMIR Pediatr Parent. 2022 Oct 19;5(4):e37448. doi: 10.2196/37448 (PMC9631170; doi:10.2196/37448)
Supplement: Multimedia Appendix 2 [file pediatrics_v5i4e37448_app2.docx]

**Online Resource 2**

List of 71 Canadian organizations, with websites that were manually searched to identify eligible resources

| **Organization or Society** | **Setting** | **Webpage** |
| --- | --- | --- |
| Canada FASD Research Network (CANFASD) | Canada | <https://canfasd.ca/> |
| Canadian Association of Midwives | Canada | <https://canadianmidwives.org/> |
| Canadian Centre on Substance Use and Addiction | Canada | <https://www.ccsa.ca/> |
| Canadian Nurses Association | Canada | <https://www.cna-aiic.ca/en> |
| Canadian Pediatric Society | Canada | <https://www.cps.ca/> |
| Government of Canada | Canada | <https://www.canada.ca/en.html> |
| Pregnancy Info | Canada | <https://www.pregnancyinfo.ca/> |
| Society of Obstetricians and Gynaecologists | Canada | <https://sogc.org/> |
| Canadian Fertility and Andrology Society Website | Canada | <https://cfas.ca/> |
| Canadian Public Health Association | Canada | <https://www.cpha.ca/> |
| National Aboriginal Council of Midwives | Canada | <https://indigenousmidwifery.ca/become-a-midwife/> |
| The MotHERS Program | Canada | <https://www.themothersprogram.ca/about> |
| Alberta Association of Midwives | Alberta | <https://www.alberta-midwives.ca/> |
| Alberta Health Services | Alberta | <https://www.albertahealthservices.ca/> |
| Government of Alberta | Alberta | <https://www.alberta.ca/index.aspx> |
| BC Women's Hospital and Health Centre | British Columbia | <http://www.bcwomens.ca/> |
| Centre of Excellence for Women's Health | British Columbia | <https://bccewh.bc.ca/> |
| First Nations Health Authority | British Columbia | <https://www.fnha.ca/> |
| Fraser Health Authority | British Columbia | <https://www.fraserhealth.ca/> |
| Government of BC | British Columbia | <https://www2.gov.bc.ca/gov/content/home> |
| HealthLink BC | British Columbia | <https://www.healthlinkbc.ca/> |
| Midwives association of British Columbia | British Columbia | <https://www.bcmidwives.com/> |
| Perinatal Services BC | British Columbia | <http://www.perinatalservicesbc.ca/> |
| Government of Manitoba | Manitoba | <https://www.gov.mb.ca/> |
| Midwives Association of Manitoba | Manitoba | <http://midwivesofmanitoba.ca/> |
| Government of New Brunswick | New Brunswick | <https://www2.gnb.ca/> |
| Public Health Association of NB and PEI | New Brunswick, Prince Edward Island | <http://www.nbpeipublichealth.ca/> |
| Association of midwives of newfoundland and Labrador | Newfoundland | <http://www.amnl.ca/> |
| Government of Newfoundland and Labrador | Newfoundland | <https://www.gov.nl.ca/> |
| Government of NWT | North West Territories | <https://www.gov.nt.ca/> |
| Association of Nova Scotia Midwives | Nova Scotia | <https://www.novascotiamidwives.ca/> |
| Government of Nova Scotia Department of Health and Wellness | Nova Scotia | <https://novascotia.ca/dhw/> |
| Nova Scotia Health Authority | Nova Scotia | [http://www.nshealth.ca/#](http://www.nshealth.ca/) |
| Reproductive Care Program of Nova Scotia | Nova Scotia | <http://rcp.nshealth.ca/> |
| Government of Nunavut | Nunavut | <https://www.gov.nu.ca/> |
| Algoma Public Health Unit | Ontario | <http://www.algomapublichealth.com/> |
| Best Start - Health Nexus | Ontario | <https://en.beststart.org/> |
| Brant County Health Unit | Ontario | <http://www.bchu.org/> |
| Centre for Addiction and Mental Health | Ontario | <http://www.camh.ca/> |
| Champlain Maternal Newborn Regional Program | Ontario | <http://www.cmnrp.ca/en/cmnrp/Home_p2974.html> |
| Chatham-Kent Health Unit | Ontario | <http://ckphu.com/> |
| Durham Region Health Department | Ontario | <https://www.durham.ca/en/health-and-wellness/health-and-wellness.aspx> |
| Eastern Ontario Health Unit | Ontario | <http://www.eohu.ca/> |
| Grey Bruce Health Unit | Ontario | <http://www.publichealthgreybruce.on.ca/> |
| Haldimand-Norfolk Health Unit | Ontario | <http://www.hnhu.org/> |
| Haliburton, Kawartha, Pine Ridge District Health Unit | Ontario | <http://www.hkpr.on.ca/> |
| Halton Region Health Department | Ontario | <https://www.halton.ca/For-Residents/Public-Health> |
| Hamilton Public Health Services | Ontario | <http://www.hamilton.ca/publichealth> |
| Hastings and Prince Edward Counties Health Unit | Ontario | <https://hpepublichealth.ca/> |
| Huron Perth District Health Unit | Ontario | <http://www.hpph.ca/> |
| Kingston, Frontenac and Lennox & Addington Public Health | Ontario | <http://www.kflapublichealth.ca/> |
| Lambton Public Health (Ontario) | Ontario | <http://www.lambtonhealth.on.ca/> |
| Leeds, Grenville and Lanark District Health Unit | Ontario | <http://www.healthunit.org/> |
| London Health Sciences Centre | Ontario | <https://www.lhsc.on.ca/maternal-newborn-care/welcome-to-maternal-newborn-care-0> |
| Middlesex-London Health Unit | Ontario | <http://www.healthunit.com/> |
| Niagara Region Public Health Department | Ontario | <http://www.niagararegion.ca/health> |
| North Bay Parry Sound District Health Unit | Ontario | <http://www.myhealthunit.ca/> |
| Northwestern Health Unit | Ontario | <http://www.nwhu.on.ca/> |
| Ontario Association of Midwives | Ontario | <https://www.ontariomidwives.ca/> |
| Ottawa Public Health | Ontario | <http://www.ottawapublichealth.ca/> |
| Peel Public Health | Ontario | <http://www.peelregion.ca/health/> |
| Peterborough Public Health | Ontario | <http://www.peterboroughpublichealth.ca/> |
| Porcupine Health Unit | Ontario | <http://www.porcupinehu.on.ca/> |
| Provincial Council for Maternal and Child Health | Ontario | <https://www.pcmch.on.ca/> |
| Public Health Ontario | Ontario | <https://www.publichealthontario.ca/> |
| Region of Waterloo, Public Health | Ontario | <http://www.region.waterloo.on.ca/ph> |
| Renfrew County and District Health Unit | Ontario | <http://www.rcdhu.com/> |
| Simcoe Muskoka District Health Unit | Ontario | <http://www.simcoemuskokahealth.org/> |
| Southwestern Public Health | Ontario | <https://www.swpublichealth.ca/> |
| Sudbury & District Health Unit | Ontario | <https://www.phsd.ca/> |
| Sunnybrook Health Sciences Centre | Ontario | <https://sunnybrook.ca/> |
| Thunder Bay District Health Unit | Ontario | <http://www.tbdhu.com/> |
| Timiskaming Health Unit | Ontario | <http://www.timiskaminghu.com/> |
| Toronto Public Health | Ontario | <http://www.toronto.ca/health/index.htm> |
| Wellington-Dufferin-Guelph Public Health | Ontario | <http://www.wdgpublichealth.ca/> |
| Windsor-Essex County Health Unit (Ontario) | Ontario | <https://www.wechu.org/> |
| York Region Public Health Services | Ontario | <http://www.york.ca/wps/portal/yorkhome/health/> |
| Association of Ontario Midwives | Ontario | <https://www.ontariomidwives.ca/> |
| [Monarch Maternal and Newborn Health](http://www.monarchcentre.ca/) | Ontario | <http://www.monarchcentre.ca/> |
| OMAMA | Ontario | <https://www.omama.com/en/index.asp> |
| Ontario Prenatal Education | Ontario | <https://www.ontarioprenataleducation.ca/medications-drugs/> |
| Prenatal Screening Ontario | Ontario | <https://www.prenatalscreeningontario.ca/en/pso/index.aspx> |
| Mothercraft | Ottawa, Ontario | <http://mothercraft.com/> |
| Ottawa Birth and Wellness Centre | Ottawa, Ontario | <https://ottawabirthcentre.ca/> |
| The Royal | Ottawa, Ontario | <https://www.theroyal.ca/> |
| Government of Prince Edward Island | Prince Edward Island | <https://www.princeedwardisland.ca/en> |
| Encadrement du cannabis au Quebec | Quebec | <https://encadrementcannabis.gouv.qc.ca/> |
| Quebec.ca | Quebec | <https://www.quebec.ca/en/> |
| Regroupement Les Sages‑femmes du Quebec | Quebec | <https://www.rsfq.qc.ca/> |
| Santé Montreal | Quebec | <https://santemontreal.qc.ca/en/> |
| Government of Saskatchewan | Saskatchewan | <https://www.saskatchewan.ca/> |
| Midwives Association of Saskatchewan | Saskatchewan | <https://www.saskatchewanmidwives.com/> |
| Government of Yukon | Yukon | <http://www.gov.yk.ca/> |
