# Supplementary material for: Canadian Resources on Cannabis Use and Fertility, Pregnancy, and Lactation: Scoping Review
Source: JMIR Pediatr Parent. 2022 Oct 19;5(4):e37448. doi: 10.2196/37448 (PMC9631170; doi:10.2196/37448)
Supplement: Multimedia Appendix 3 [file pediatrics_v5i4e37448_app3.docx]

**Online Resource 3**

Full database search strategy

*Database searching*

The following databases were searched by a health sciences librarian (LS) during the electronic component of the systematic review: Medline and Medline in Process via Ovid (1946 – November 25, 2020), Embase Classic + Embase via Ovid (1947 – November 25, 2020), ERIC via Ovid (1965 – September 2020), CINAHL via EBSCOHost (1981 – November 30, 2020) and Education Source via EBSCOHost (1880 – November 30, 2020). A search strategy was developed in Medline, and then translated into the other databases, as appropriate (see Appendix 1). The search strategy was peer reviewed using the Peer Review of Electronic Search Strategies (PRESS) guidelines by AH. All databases were searched from January 2010 to November 30, 2020. There were no publication restrictions. All references were entered into an Endnote file for processing (n = 267).

**Database searches (numbers for PRISMA flowchart):**

Medline in Process and Medline (via Ovid): n = 61

Embase (via Ovid): n = 137
 ERIC (via Ovid): n = 0

CINAHL (via EBSCOHost): n = 60

Education Source (via EBSCOHost): n = 9

Total: n = 267

Duplicates: n = 70

Total with duplicates removed: n = 197

**Medline**
1. exp pregnancy/

2. exp pregnancy trimesters/

3. pregnan*.tw.

4. prenatal care/

5. postnatal care/

6. perinatal care/

7. Pregnant Women/

8. (prenatal or pre-natal or postnatal or post-natal or perinatal or peri-natal or antenatal or ante-natal or postpartum or post-partum or parturition or puerperium or gestation or gestating or gestate).tw.

9. exp Postpartum Period/

10. exp Breast Feeding/

11. Milk, Human/

12. (breastfeeding* or breast feeding* or lactation or lactating or breast milk or breastmilk or human milk or chest milk or chestmilk).tw.

13. exp Fertility/

14. exp Fertility Agents/

15. Fertility Preservation/

16. Involuntary Fertility Control/

17. exp Infertility/

18. exp Fertilization/

19. (fertil* or infertil* or fecund* or subfecund* or steril* or subfertilit* or sub-fertilit*).tw.

20. (fertiliz* or conception* or conceiv*).tw.

21. or/1-20

22. Cannabis/

23. exp "Marijuana Use"/

24. exp cannabinoids/ or Medical marijuana/ or Marijuana abuse/

25. (cannabi* or marijuana* or marihuana* or bhang or bhangs or ganja or ganjas or hashish or hashishs or hash or hemp or hemps or ganjah or cannador or charas or kush or weed or dronabinol or cannabichromene or THC or CBD or nabilone or tetrahydrocannabivarin or ganka or marinol or cesamet or dexanabinol or sativex or "HU211" or "HU 211" or indica or syndros or cannabinoid or cannabinoids or cannabidiol or nabiximols or namisol).tw.

26. ((blunt or blunts) adj2 smok*).tw.

27. or/22-26

28. exp Canada/

29. (canada or alberta or british columbia or yukon or northwest territories or nunavut or manitoba or saskatchewan or ontario or quebec or newfoundland or prince edward island or nova scotia or new brunswick).tw.

30. Canada.cp.

31. 28 or 29 or 30

32. 21 and 27 and 31

33. limit 32 to yr="2010 -Current"

**Embase**

1. exp pregnancy/

2. puerperium/

3. pregnant women/

4. pregnan*.tw.

5. prenatal care/

6. postnatal care/

7. perinatal care/

8. (prenatal or pre-natal or postnatal or post-natal or perinatal or peri-natal or antenatal or ante-natal or postpartum or post-partum or parturition or puerperium or gestation or gestating or gestate).tw.

9. intrapartum care/

10. exp breast feeding/ or breast milk/

11. (breastfeeding* or breast feeding* or lactation or lactating or breastmilk or breast milk or human milk).tw.

12. exp fertility/

13. exp fertility parameters/

14. fertility preservation/

15. involuntary fertility control/

16. (fertil* or infertil* or fecund* or subfecund* or steril* or subfertilit* or sub-fertilit*).tw.

17. (fertiliz* or conception* or conceiv*).tw.

18. or/1-17

19. cannabis/

20. "cannabis use"/

21. cannabis addiction/

22. medical cannabis/

23. (cannabi* or marijuana* or marihuana* or bhang or bhangs or ganja or ganjas or hashish or hashishs or hash or hemp or hemps or ganjah or cannador or charas or kush or weed or dronabinol or cannabichromene or THC or CBD or nabilone or tetrahydrocannabivarin or ganka or marinol or cesamet or dexanabinol or sativex or "HU211" or "HU 211" or indica or syndros or cannabinoid or cannabinoids or cannabidiol or nabiximols or namisol).tw.

24. ((blunt or blunts) adj2 smok*).tw.

25. or/19-24

26. exp Canada/

27. (canada or alberta or british columbia or yukon or northwest territories or nunavut or manitoba or saskatchewan or ontario or quebec or newfoundland or prince edward island or nova scotia or new brunswick).tw.

28. canada.cp.

29. 26 or 27 or 28

30. 18 and 25 and 29

**ERIC**

1. exp pregnancy/

2. pregnant students/

3. pregnan*.tw.

4. (prenatal or pre-natal or postnatal or post-natal or perinatal or peri-natal or antenatal or ante-natal or postpartum or post-partum or parturition or puerperium or gestation or gestating or gestate).tw.

5. (breastfeeding* or breast feeding* or lactation or lactating or breast milk or breast milk or human milk).tw.

6. (fertil* or infertil* or fecund* or subfecund* or steril* or subfertilit* or sub-fertilit*).tw.

7. (fertiliz* or conception* or conceiv*).tw.

8. or/1-7

9. Marijuana/

10. (cannabi* or marijuana* or marihuana* or bhang or bhangs or ganja or ganjas or hashish or hashishs or hash or hemp or hemps or ganjah or cannador or charas or kush or weed or dronabinol or cannabichromene or THC or CBD or nabilone or tetrahydrocannabivarin or ganka or marinol or cesamet or dexanabinol or sativex or "HU211" or "HU 211" or indica or syndros or cannabinoid or cannabinoids or cannabidiol or nabiximols or namisol).tw.

11. ((blunt or blunts) adj2 smok*).tw.

12. or/9-11

13. (canada or alberta or british columbia or yukon or northwest territories or nunavut or manitoba or saskatchewan or ontario or quebec or newfoundland or prince edward island or nova scotia or new brunswick).tw.

14. 8 and 12 and 13

**CINAHL**

| **Search Terms** | **Search Options** |
| --- | --- |
| S23 | S14 AND S17 AND S22 |
| S22 | S10 OR S19 OR S20 OR S21 |
| S21 | (fertiliz* or conception* or conceiv*) |
| S20 | (fertil* or infertil* or fecund* or subfecund* or steril* or subfertilit* or sub-fertilit*) |
| S19 | (MH "Fertility+") OR (MH "Fertility Preservation") OR (MH "Fertilization+") |
| S18 | S10 AND S14 AND S17 |
| S17 | S15 OR S16 |
| S16 | (canada or alberta or british columbia or yukon or northwest territories or nunavut or manitoba or saskatchewan or ontario or quebec or newfoundland or prince edward island or nova scotia or new brunswick) |
| S15 | (MH "Canada+") |
| S14 | S11 OR S12 OR S13 |
| S13 | ((blunt or blunts) N2 smok*) |
| S12 | (cannabi* or marijuana* or marihuana* or bhang or bhangs or ganja or ganjas or hashish or hashishs or hash or hemp or hemps or ganjah or cannador or charas or kush or weed or dronabinol or cannabichromene or THC or CBD or nabilone or tetrahydrocannabivarin or ganka or marinol or cesamet or dexanabinol or sativex or "HU211" or "HU 211" or indica or syndros or cannabinoid or cannabinoids or cannabidiol or nabiximols or namisol) |
| S11 | (MH "Cannabis+") OR (MH "Medical Marijuana") |
| S10 | S1 OR S2 OR S3 OR S4 OR S5 OR S6 OR S7 OR S8 OR S9 |
| S9 | (breastfeeding* or breast feeding* or lactation or lactating or breast milk or breast milk or human milk) |
| S8 | (prenatal or pre-natal or postnatal or post-natal or perinatal or peri-natal or antenatal or ante-natal or postpartum or post-partum or parturition or puerperium or gestation or gestating or gestate) |
| S7 | pregnan* |
| S6 | (MH "Perinatal Care") OR (MH "Intrapartum Care+") OR (MH "Prenatal Care") |
| S5 | (MH "Postnatal Period+") |
| S4 | (MH "Milk, Human") |
| S3 | (MH "Breast Feeding") |
| S2 | (MH "Expectant Mothers") |
| S1 | (MH "Pregnancy+") OR (MH "Pregnancy, Multiple+") OR (MH "Pregnancy Trimesters+") OR (MH "Pregnancy, Unplanned") OR (MH "Pregnancy, Unwanted") |

**Education Source**

| **Search Terms** | **Search Options** |
| --- | --- |
| S13 | S7 AND S8 AND S12 |
| S12 | S4 OR S10 OR S11 |
| S11 | (fertiliz* or conception* or conceiv*) |
| S10 | (fertil* or infertil* or fecund* or subfecund* or steril* or subfertilit* or sub-fertilit*) |
| S9 | S4 AND S7 AND S8 |
| S8 | (canada or british columbia or alberta or saskatchewan or manitoba or ontario or quebec or new brunswick or nova scotia or prince edward island or newfoundland or yukon or northwest territories or nunavut) |
| S7 | S5 OR S6 |
| S6 | ((blunt or blunts) N2 smok*) |
| S5 | (cannabi* or marijuana* or marihuana* or bhang or bhangs or ganja or ganjas or hashish or hashishs or hash or hemp or hemps or ganjah or cannador or charas or kush or weed or dronabinol or cannabichromene or THC or CBD or nabilone or tetrahydrocannabivarin or ganka or marinol or cesamet or dexanabinol or sativex or "HU211" or "HU 211" or indica or syndros or cannabinoid or cannabinoids or cannabidiol or nabiximols or namisol) |
| S4 | S1 OR S2 OR S3 |
| S3 | (breastfeeding* or breast feeding* or lactation or lactating or breast milk or breast milk or human milk) |
| S2 | (prenatal or pre-natal or postnatal or post-natal or perinatal or peri-natal or antenatal or ante-natal or postpartum or post-partum or parturition or puerperium or gestation or gestating or gestate) |
| S1 | pregnan* |
