# Supplementary figures and images for: Canadian Resources on Cannabis Use and Fertility, Pregnancy, and Lactation: Scoping Review
Source: JMIR Pediatr Parent. 2022 Oct 19;5(4):e37448. doi: 10.2196/37448 (PMC9631170; doi:10.2196/37448)

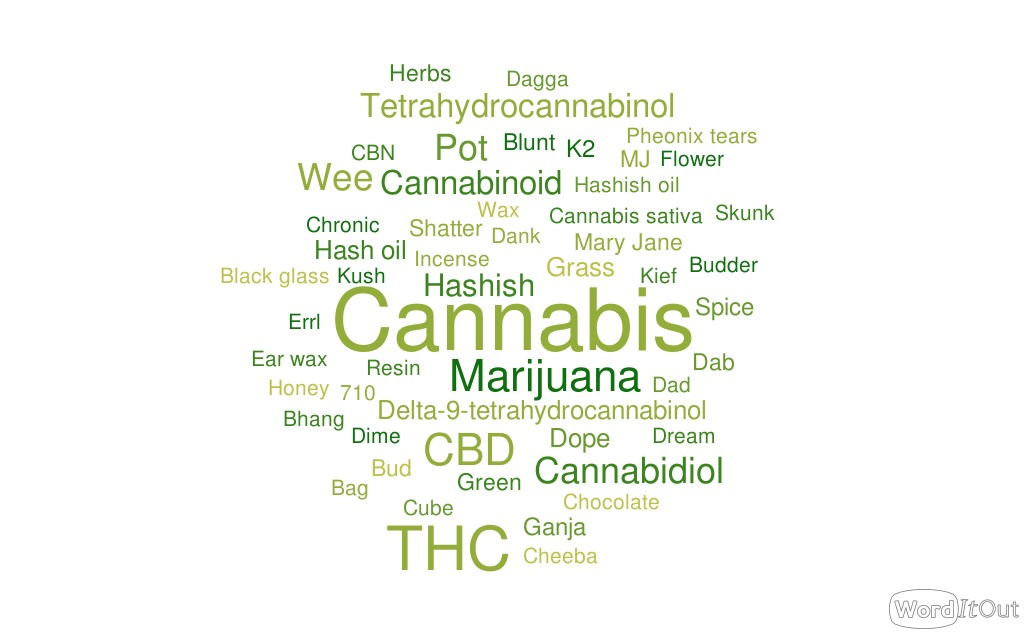

Supplement: Multimedia Appendix 5 [file pediatrics_v5i4e37448_app5.png]
